# Voyages in Vacation.—XIV

**Published:** 1889-01-12

**Authors:** 


### VOYAGES IN VACATION.—XIV.

By One in Search of Restful Restoratives.

*(Continued from page 183.)*

### THE KREMLIN, MOSCOW.

Everybody has heard of the Kremlin, situated in the heart of Moscow, and possessing an historic interest almost, if not quite, as great as any object to be met with in modern Europe. It is surrounded by a castellated wall upwards of 7,000 feet in circumference, surmounted by eighteen towers, and pierced by five gates. Of these gates the most famous is the Spaski, or Holy Gate, over which is a picture of our Saviour, said to have been brought from Smolenski by the Tzar Alexis, in 1647. So deep is the reverence in which this gate is held by the orthodox, that anyone omitting to uncover when passing through it was liable to punishment. The Emperor himself invariably uncovers when passing through Holy Gate, and at the present date, all travellers who are wise will conform to this custom. The River Moskwa running east and west forms the southern boundary of the Kremlin, the best view of which is obtainable from the handsome bridge which crosses it a short distance off. In the centre of the Kremlin is a small square, known as Cathedral Place, at the four corners of which stand the most sacred and famous buildings in all Russia. In the north-west corner is the Cathedral of the Assumption, in which all the Russian Tzars from Ivan the Terrible to the present Emperor have been crowned. In the south-west is the Church of the Annunciation, where the reigning house worship when in residence at Moscow, and where the French soldiers stabled their horses—a deliberate act of defilement known to be particularly distressing to the Russian people. On the south-east is the Cathedral of the Archangel Michael, where all the old Tzars are buried, their successors being now interred in the Church of St. Peter and St. Paul, within the Citadel of St. Petersburg. In the north-west corner stands the celebrated Tower of Ivan, already alluded to, from which the most impressive view of Moscow is obtainable. These churches are a favourite resort of pilgrims, and, though relatively small, they are full of relics, and present many features of the deepest interest to the antiquary and the historian. It was curious to note the misery and wretchedness of the Russian peasantry and their superstition, as marked by the pilgrims we met in the churches during our visit. Abominably filthy, with ill-kempt hair, ragged clothing, and every attribute of grinding poverty, the reverence they displayed in the presence of relics, and their evident sincerity before the sacred shrines must have impressed those who saw it. They were, no doubt, specimens of the inhabitants of the villages, a description of which was given in a former article,* and their presence and appearance made us wish for an opportunity to try and arouse the sympathies of the Russian authorities, and so to alleviate much of the avoidable suffering which they have perforce to endure in their present neglected and uncared-for condition.

The synodal buildings, formerly the patriarchs' house, situated within the Kremlin, contain many ancient robes, ornaments, church vessels and plate, some of which date back to the year 1300. Here is the remarkable Sakko, presented by Ivan the Terrible, an ancient robe of lavish magnificence, enriched by innumerable rubies, emeralds, diamonds, garnets, and other precious stones, the whole weighing, it is said, 54 lbs. Amongst the mitres formerly worn by the patriarchs here preserved is one studded with large diamonds, rubies, emeralds, sapphires, and pearls, which weighs upwards of 5 lbs. Ancient charms of costly pattern and elaborate workmanship, pastoral staffs, vases, and valuable plate are met with in abundance. Here also the Holy Oil is made every three years, to supply the whole of Russia. It is prepared in great silver kettles being afterwards poured into silver jars, and distributed on application, to the bishops throughout the country. Everything employed in the preparation of the Holy Chrism is of silver, the various vessels now in use weighing altogether about 13 cwt. The oil is prepared in Martyrs' week, the Archbishop and other dignitaries of the church lighting the fire and attending to the boiling for three days and nights. The finest Florentine oil is used, thirteen spices of great value being added, and a few drops of the oil from Mary Magdalene's flask. This flask is preserved in a small chapel, being placed upon a table covered with a gold-worked cloth, and is shaped like a water-bottle. The flask is believed to have been used by Mary Magdalene when anointing the Saviour's feet, and to contain some of the oil, mingled with her own tears, with which she washed His feet. Hence the great veneration in which it is held, and the reason for the refusal to admit visitors to the chapel, which is considered holy ground.

The great palace of Bolshoi Dvorets, an odd mixture of various periods of architecture, containing 700 rooms, and built between 1838—49, next claimed our attention. It is not possible to give a full description of the palace, but a few of the more interesting features may be pointed out. Amongst these is the square hall, said to have been built in 1491, and since restored by Nicholas I., called the Grano Vitaya Palato, where the Emperor ascends to an elaborate throne, after his coronation, wearing all the Imperial insignia, and where he dines with his nobles, reigning monarchs alone being permitted to sit at the same table with him. At one end is a window communicating with a room which the members of the Imperial family occupy during this ceremony, as they are excluded from the hall in accordance with ancient etiquette, which is still enforced. We can well imagine that the Imperial Emperors, being mortal, must be heartily glad when this function is over. Another portion of the building, called the Terem, will interest most visitors and especially ladies, as it was occupied in old days by the Tsaritsa and her children. At the Treasury, in the Round Room, many interesting relics, including most of the regalia, are preserved. Here is shown the ivory throne of a late Emperor of Constantinople, with elaborate carving representing the Labors of Orpheus and the Legend of Thrace. Here also is the throne used by the Tzar Alexis, which is studded with more than two thousand diamonds and other precious stones, and which is still used by the Empress at her coronation. Another throne of great interest is literally covered with small turquoise, said to number at least nine thousand, There are also many thrones used by various Tzars, most of which are bedecked with precious stones. The crown of Peter the Great is probably the most magnificent of all the crowns, as it contains some 900 diamonds in addition to very many rubies and emeralds. The coronation robes with the boots of Peter the First, and those of other emperors are also interesting, being in many instances, as can well be imagined, quite unique. In another part of the palace the old carriages of the Court of Moscow are exhibited, one of which was presented by Queen Elizabeth to the reigning Tzar in her day. In the same apartment is a small carriage used by Peter I. when a child, and also two camp bedsteads which Napoleon brought with him, and which were afterwards taken during the retreat from Moscow. Finally, we must not omit to mention the Armoury, containing many stands of arms and armour kept in the most perfect order, and of great interest. In the Treasury are preserved the innumerable presents given to the Russian houses by foreign potentates in all parts of the world, many of them being fine specimens of the artistic work produced by eastern and western countries in ancient times.

Outside the Kremlin is the cathedral of St. Basil, which presents a grotesque appearance. It has eleven towers, each exhibiting distinct characteristics of color or design, or both. The cathedral has eleven chapels, one beneath each dome, which are approached by a number of irregular passages, very confusing to the visitor. A curious tradition, now stated to be untrue, declares the church to be the work of an Italian architect. When the building was completed, Ivan the Terrible was so greatly pleased with it, rightly believing it to be unique, that he is said to have invited the architect to dinner. After dinner, he complimented him upon his work, and ordered his eyes to be put out in order that he might be unable to design any similar building for erection elsewhere.

*(To be continued.)*

* *Vide* The Hospital, No. 117, page 183.

